# Generalized Behavior Framework for Mobile Robots Teaming With Humans in Harsh Environments

**DOI:** 10.3389/frobt.2022.898366

**Published:** 2022-06-29

**Authors:** Oliver Avram, Stefano Baraldo, Anna Valente

**Affiliations:** Automation, Robotics and Machines Laboratory, Department of Innovative Technologies, Institute of Systems and Technologies for Sustainable Production, University of Applied Sciences and Arts of Southern Switzerland (SUPSI), Viganello, Switzerland

**Keywords:** robotics, human-robot interaction (HRI), behavior trees (BT), harsh environments, human-aware task planning

## Abstract

Industrial contexts, typically characterized by highly unstructured environments, where task sequences are difficult to hard-code and unforeseen events occur daily (e.g., oil and gas, energy generation, aeronautics) cannot completely rely upon automation to substitute the human dexterity and judgment skills. Robots operating in these conditions have the common requirement of being able to deploy appropriate behaviours in highly dynamic and unpredictable environments, while aiming to achieve a more natural human-robot interaction and a broad range of acceptability in providing useful and efficient services. The goal of this paper is to introduce a deliberative framework able to acquire, reuse and instantiate a collection of behaviours that promote an extension of the autonomy periods of mobile robotic platforms, with a focus on maintenance, repairing and overhaul applications. Behavior trees are employed to design the robotic system’s high-level deliberative intelligence, which integrates: social behaviors, aiming to capture the human’s emotional state and intention; the ability to either perform or support various process tasks; seamless planning and execution of human-robot shared work plans. In particular, the modularity, reactiveness and deliberation capacity that characterize the behaviour tree formalism are leveraged to interpret the human’s health and cognitive load for supporting her/him, and to complete a shared mission by collaboration or complete take-over. By enabling mobile robotic platforms to take-over risky jobs which the human cannot, should not or do not want to perform the proposed framework bears high potential to significantly improve the safety, productivity and efficiency in harsh working environments.

## 1 Introduction

The sector of Maintenance, Repair and Overhaul (MRO) has reached a value of 686.6 Billion USD in 2020, and is expected to grow with a CAGR of 2.3% until 2027, reaching a total value of 787.2 USD (Expert Market Research, 2020). In this huge and diversified scenario of activities, inspection and repair, especially offshore, are still entirely human based, since the employed workforce is extremely skilled in running manufacturing tasks, but also in effectively undertaking complex decisions in very critical operating scenarios. Men and women executing work tasks in harsh conditions push their physical and psychological resources to their limits, therefore exposing them to high risks for their wellbeing. As a result, the world of MRO assists yearly to the death of 3.5 thousand expert human operators as a result of accidents and 3.3 million non-fatal injuries of various nature (Eurostat, 2020). This is neither related to poor safety measures nor to superficial human behavior: harsh environment and unforeseen events, including unpredictable faults and malfunctions, dramatically raise the level of danger.

The recent advances of robotics and artificial intelligence are suggesting that, in this kind of contexts, the introduction of a new generation of service and maintenance robots could be the ideal way to safeguard the health of workers, if they could achieve a comparable quality in performing maintenance operations. Nonetheless, this would hardly be enough to substitute a human worker, as the human decision capacity, especially in unstructured environments, will still be unreachable for years by AI. The best trade-off between safety, reliability and decision capacity is nowadays widely accepted as the human-robot interaction, as testified by the increasing number of collaborative and environment-aware robots proposed by manufacturers (Grau et al., 2021).

As mentioned, not only harsh work places are hazardous for the physical health of workers, but they are also sources of mental stress and fatigue, which could lead to anxiety, decrease of attention and mistakes. Moreover, human beings naturally communicate not only by assertion, but also through their behavior, attitude, expression, etc.,. Therefore, it is of capital importance that robots conceived as teammates for harsh environments are able to detect and to interpret a wide range of indirect signals from human workers (e.g., voice tone, facial expression, posture) and their relation to their actual context, to be able to work with them and to preserve their health. This leads to the necessity of developing robot assistants that implement some notion of *artificial empathy (AE)* (Yalcin and Di Paola, 2018). In this work, AE will be intended as the capacity to detect in humans feelings of either physiological or emotional nature, which could indicate or preempt a danger either for their health, for the success of a task or for the plant functioning, and to formulate a proper reaction to them.

In addition to understanding some notion of worker status (health and mental), of course a mobile service robot should provide a service in the context of an unstructured environment, like an offshore oil platform, a wind farm or a gas pipeline. Such operations may be for example the inspection of a difficult-to-reach surface to identify damages, cleaning and de-rusting, re-coating, closure of leaks by welding or additive manufacturing, logistics, and others. Examples of robots with such capabilities are gradually appearing both in research (Schmidt and Berns, 2013; Gitardi et al., 2021) and as commercial products (Anybotics, 2022; Eddify, 2022; Rope Robotics, 2022). These operations must be planned, supervised and validated by a human worker, who has the necessary experience and decision capacity to guide them to proper execution, therefore also during this kind of tasks, empathic communication has a central role. The whole set of the aforementioned communication and collaboration tasks are called Human-Robot Interaction (HRI).

Starting from these premises, this work is motivated by the need of a holistic approach to study the HRI in harsh environments with the focus on the formulation of adapted robotic behaviors directed towards the alleviation of the perceived stress factors and human exposure to dangerous situations.

The main novelty is the use of Behavior Trees (BT) to design a comprehensive HRI framework that enables the robot to select and perform actions adapted to the constraints imposed by the presence of humans with different health and emotional states, requirements and preferences. The framework leverages an extended set of functional robotic capabilities (i.e., navigation, perception of human activities, communication, emotion recognition, task risk assessment, planning and execution of shared MRO tasks) to address the complexity of human-robot joint decision making dynamics during operation in harsh contexts. The management of the human robot interaction is considered as an integral part of the high-level robot’s control architecture aiming to identify human key behavioral factors affecting the robot’s decisional process.

These factors are tackled in various contexts: the robot ascertains the current working situation involving the human “through the eye of an external observer”; the robot grasps psychosocial factors affecting human health and behavior (e.g., in addition to physical difficulties of handling certain tasks, an excessive human emotional burden could lead to irreversible losses); the robot takes a task-based perspective to identify, assess and mitigate potential risks with focus on the manufacturing human-robot collaborative process; the robot assigns roles and responsibilities and proposes alternative courses of actions to alleviate the difficulty of tasks to be handled by the humans (i.e., through human-robot shared plan).

In order to meet teammates’ needs and requirements in HRI, the proposed framework provides a BT interface facilitating the authoring of robot’s behaviors grouped into two main HRI phases:

• The *Safety HRI* behavior tree has the aim to support a human agent by observing her/his psycho-physical status, processing the information and deliberating an immediate decision.
• The *MRO HRI* behavior tree determines the main environmental and situational risk conditions for the operator, generating an *empathy map*, and based on an *interdependence analysis* processes a task sequence to propose feasible alternative workload distributions between human and robot, with the aim of minimizing hazards and task failures.

The paper is structured as follows. Section 2 regards methodology: general presentation of behavior trees (section 2.1), main assumptions (Section 2.2), and description of the considered human-robot interaction modes (Section 2.3). Section 3 describes the proposed framework, in particular describing the behavior trees dedicated to Safety (Section 3.1) and MRO (Section 3.2). Section 4 discusses the main features of the proposed approach. Finally, Section 5 regards open points and perspectives on the implementation in a real case.

## 2 Methods

This section introduces the main methodological concepts exploited to develop the framework described in Section 3, as well as the underlying assumptions about the environment, involved human worker, robot, and their relationship in a collaborative context.

The main tool used to structure the proposed deliberative framework is the formalism of *behavior trees*, which are briefly presented in the following.

### 2.1 Behavior Trees

Behavior trees (BTs) have been introduced in AI programming for the videogame industry, obtaining great success in the last 10 years because of their flexibility and readability. Nowadays, many of the main environments dedicated to videogame programming integrate BTs (Unity, 2021; Unreal Engine, 2022), offering also visual interfaces for designing and automatically implementing them. Their usefulness has been rapidly acknowledged by researchers and developers of AI for robotics (Marzinotto et al., 2014; Ghzouli et al., 2020; Iovino et al., 2020), also recognizing their effectiveness in generalizing several other formalisms for coding hybrid dynamical systems (Colledanchise and Ögren, 2017).

A BT is a directed acyclic graph, which origins from a *root* node, splits into several branches through *control flow* nodes and is terminated by *execution* nodes, i.e., are graph leaves. Cyclically, the root node generates a tick, i.e., an enabling signal that follows a different path along the tree depending on the states and types of traversed control flow nodes. Eventually, the tick reaches execution nodes, which are dedicated to performing actual operations. When they are reached by the tick, execution nodes return to the parent node a state variable, which can have value *Success*, *Running* or *Failure*. In turn, control flow nodes collect the states of child nodes, and return their own state depending on them and on their own type. The whole BT is supported by a set of global variables, called Blackboard (BB), which are accessible to all the nodes and are dynamically modified by them.

The node types are described in Table 1. In typical visual representations of BTs, nodes are laid out from left to right depending on their priority of execution, so the term “sequentially” used in the following implies that nodes are evaluated in a well-established order, which is coherent with the graph representation.

**TABLE 1:** Main BT node types.

Category	Type	Notation	Function	Returns
Root	Root		Ticks its only child	The state returned by its child
Control flow	Selector	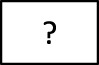	Ticks children sequentially until one returns Success or Running	Success when it receives a Success from a child
				Failure when all children return Failure
	Sequence	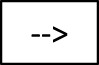	Ticks children sequentially until one returns Running or Failure	Success if all children return Success
				Running/Failure when a child returns Running/Failure
	Parallel Sequence	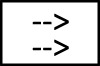	Ticks all of its children simultaneously until a child task returns failure	Success when all of its children tasks return success
				Failure when a child task returns failure
				Running otherwise
	Parallel Selector	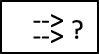	Ticks all of its children simultaneously until a child task returns success	Success when one of its children tasks returns success
				Failure when all child tasks return failure
				Running otherwise
	Decorator	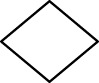	Ticks its only child only if a condition is satisfied	Depending on a function applied to a set of variables
Execution	Action	Green box	Performs operations	Depending on its purpose
	Condition	Yellow ellipse	Applies a function to determine the return state	Depending on its purpose

For a comprehensive introduction on BT, we refer readers to Colledanchise and Ögren (2018).

**FIGURE 1:**
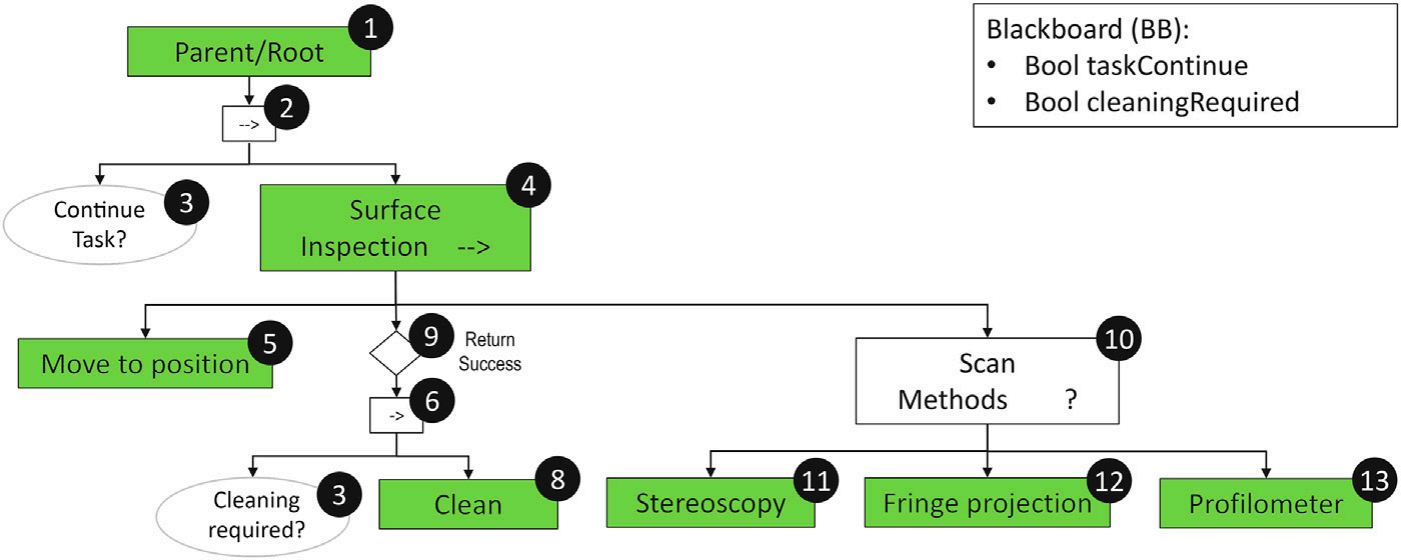
Behavior tree example. Unless an external signal stops the task (node 3), the inspection sequence executes: motion to the target area (node 5); evaluation if cleaning is required (subtree of node 9); and, finally, selection of the most effective scanning methods (subtree of node 10).

Figure 1 presents an example of BT for an inspection task of a mobile service robot.

The BT root (possibly a parent node from a BT of higher hierarchy level) ticks at a specific frequency the main inspection task node 2, which will pass the tick to one of its children, depending on the type and policy of the root node. In this example, the evaluation of the children is run sequentially with a first check on task execution clearance 3 followed by the actual surface inspection operation 4. The sequence node 2 fails if either of its children returns failure, e.g., an external signal interrupts the task (pre-condition node 3—c*ontinue Task = = false*) or the *Surface inspection* sequence fails.

The *Surface inspection* sequence 4 first moves the robot to the target position, by triggering a low-level routine. While the robot is planning and/or moving towards the target position the *Move to position* node 5 returns a *Running* state to its parent node (i.e., *Surface inspection*). If the robot is unable to reach the target position (e.g., there is no viable path, the robot has fallen over) the *Move to position* will return a *Failure* state. Once the position is reached (i.e., *Move to position* returns *Success*) the evaluation of the Surface inspection tree moves to the next child and the robot checks if a cleaning pre-treatment is required for that kind of surface (pre-condition 7—*Cleaning Required?*); in case it is required (*cleaningRequired = = true*), it ticks the *Clean* action 8, which upon completion will return *Success*. If no cleaning is required, the sequence node 6 will return failure, which will be converted in success by the *Return Success* decorator 9. After the optional cleaning, all the available surface inspection methods (11- *Steoreoscopy*, 12-*Fringe projection*, 13—*Profilometer*) are tried by the *Scan Methods* selector 10, until one succeeds.

Some of the most prominent features justifying the use of a BT representation are its *modularity*, *reactiveness* and *deliberative* structure.

Modularity can be easily achieved in BTs by implementing one activity through an independent BT, i.e., a BT that has no interactions with other nodes of the parent tree, other than receiving the tick. This feature of BTs is very important in our framework, as it allows the reuse of subtrees in other trees (Colledanchise and Ögren, 2017) to sequence the tasks of a mission with the support of a mission planner that queues BT templates and reference tasks under proper control nodes.

Intuitively, the reactiveness of a system means the formulation of an appropriate response to the various changes in the environment. In the BT case this is achieved through a repeated ticking which frequently polls the environment for its current state to better support the decision making and to make the behavior transparent and predictable (Sprague and Ögren, 2018; Biggar et al., 2020).

Deliberate action is a central issue in robotics mainly motivated by the need of endowing autonomous robot with critical capabilities to cope with a variety of environments, tasks and interactions (Ingrand and Ghallab, 2017). One such critical capability is the continual planning and deliberation addressing the agent’s ability to react to events, to update and repair its plans based on its contextual perception (Ghallab et al., 2016). BTs can also be used to create deliberative agents, where the actions are carried out in order to reach a specific goal. For instance, BT can be used as control architecture in an automated planning by enabling a reasoning process that is both hierarchical and modular and can monitor, update and extend plans while acting (Colledanchise and Ögren, 2018). Within our framework, the deliberation is enabled not only by the entire structure of the tree but also by the feedback given by dedicated deliberative subtrees (see Section 3.2).

### 2.2 Assumptions

As anticipated, the proposed behavioral framework has been developed with a focus on unstructured work places, where mobile service and maintenance robots can be effectively used to assist human work activities. The main assumptions regarding these elements are described in the following.

#### 2.2.1 Environment

The target applications are large work plants (e.g., more than 1,000 m^2^), potentially laid out on more than one floor, comprising a multitude of fixed and moving obstacles. The plant map is known and available to the robot, although variations from nominal can be present at any time because of moving obstacles (workers, other robots, transports) or changed landscape. We consider the environment reachable by the robot in navigation mode as bidimensional, or at least easily representable by a multi-layer 2D map, to support multiple decks and irregular but easily traversable terrains. More general cases, where considering environments as intrinsically 3D may be mandatory also during navigation (e.g., with reference to drones), could be also accommodated by the proposed approach, but they are not treated for simplicity.

#### 2.2.2 Human Workers

Human workers considered in this framework are capable of moving by walking and communicating by speaking and using gestures. Their phrases are assumed to be fully understandable by the robot, at least the ones that are of interest for its operations. Of course, using Natural Language Processing (NLP) to convey commands to a robot in a general, possibly harsh environment is an open research branch, but it is considered out of scope for this work.

The facial expression of a human is considered as an important indicator of the worker’s health and emotional status, although it may not be always available to the robot, not only because of visual impairing, but also due to helmets, masks etc. For this reason, it is important to address multimodal agent status recognition, possibly acquiring additional information from voice tone, physiological sensors and body language.

The human is considered to have a much superior judgement capacity, compared to the robot, when in a favorable emotional condition (e.g., happiness, relaxedness, focus), and can be put into discussion only when high danger is associated to a heavily altered emotional or health state (e.g., nervous, unresponsive, injured, incapacitated).

#### 2.2.3 Robot

The considered robot is mobile and autonomous, i.e. it is able to navigate between two points on the available plant map without human guidance, automatically avoiding unexpected and moving obstacles. To do this, the robot is equipped with a locomotion system (e.g., wheels or legs) and proper sensors.

The robot is equipped with a vision system, which serves multiple functions. Other than supporting the navigation, as mentioned before, it is used to detect humans, identify them and classify their emotional and health state. Moreover, it is used in inspection tasks, which consist in commanding the robot to reach a location, search for damages, leaks or other visual features that require intervention, and to perform the actual inspection (e.g., take pictures of the area of interest, measure a crack size). This can happen either in an unsupervised or supervised manner, with the operator looking through the robot’s eyes by a screen and giving directions about where to move. In addition to the vision system the robot can carry other inspection systems (e.g., profilometer, 3D scanner).

The robot is able to understand human vocal commands, at least within a pre-defined set of phrases, and to speak itself, at least by a pre-defined set of phrases. If available and allowed by factory policies, the robot may also connect to health-tracking devices worn by a nearby operator and read signals like heartbeat frequency or electrodermal activity.

Internally, the robot stores important information to operate in an MRO environment: plant map, worker identifiers and associated features, manufacturing operations programs, and others.

A remote connection to a *base*, i.e., a plant operations control center should always be available: primarily, to send emergency signals for injured workers; secondly, to update and validate shared work plans, receive new instructions, ask for equipment, etc.

Finally, the considered robot is able to perform one or more MRO activities. This term is used here to describe any maintenance, repair or worker support operation that requires a tool and a physical interaction of the robot with the environment, other than navigation. In this respect, the robot configuration consists of a mobile platform equipped with a robotic arm, tool changer and is able to carry several manufacturing tools.

Table 2 resumes a non-exhaustive list of robotic capabilities jointly with their enabling technologies, relevant for the proposed framework.

**TABLE 2:** Required robot capabilities.

Capability	Description	Enabling technologies
Locomotion	The robot moves along a defined path	Wheels/crawlers/legs
Autonomous navigation	The robot localizes its own position, defines navigation paths and runs them. Detects and avoids unexpected and moving obstacles	LIDAR, GPS, Time Of Flight (TOF), Stereoscopic vision
Object recognition	The robot recognizes object of interest in the surrounding landscape, in particular humans and mission-critical tools	Stereoscopic vision, TOF, vision processing, machine learning
Remote communication	The robot communicates with the control base, either continuously or when necessary, to receive/send commands, data and calls for support or rescue	5G, Wi-Fi
Speech recognition	The robot recognizes speech during human-robot interaction, understands key phrases and parses them as commands	Microphone, NLP
Face recognition	The robot recognizes face expressions and identifies emotions from them	Vision processing, machine learning
Posture recognition	The robot is able to classify the posture of humans (e.g. as standing, sitting, lying), and their movement type (still, working, walking, unnatural/injured)	Vision processing, machine learning
Defect recognition	The robot recognizes common types of defects (e.g. leaks, rust, bumps, cracks, abrasions, burning)	Vision processing, 3D scanner/profilometer
Speech generation	The robot generates words and possibly speaks with human-like voice in human-robot interaction	Speakers, NLP
Manufacturing operation #	A specific type of manufacturing operation	Manufacturing tool, tool selector, vision processing, CAM engine for repair, force/torque sensor
Environmental conditions sensing	The robot takes measurements of the outdoor environment	Wind, temperature, humidity sensors
Life sign checking	The robot can connect with available health-tracking devices worn by the present work mate and analyse them to assess health and emotional state	Bluetooth/Wi-Fi, heart rate sensor, skin capacity sensor

### 2.3 Human Robot Team Configuration and Interaction

Trends in the MRO industry suggest a growing need for enhanced robotic automation in order to conduct operations in harsh environments, as well as to improve the safety and efficiency of workers in existing facilities (Heyer 2010). The aim of using robots in harsh environments and the need for higher degree of autonomy and deliberative skills during the interaction with the human promotes a paradigm shift from seeing the robot as an instrument to seeing it as a teammate. In this role there are higher expectations for the robot to provide proactive assistance in the planning and execution of the work activities. Nevertheless, more work is needed to smoothly interface humans in different roles and robots toward a joint team working on a common goal. The robot, seen as an equal peer complying with the team’s rules, should also be capable of varying its level of autonomy and involvement depending on environment and situation. Recently, research has been using the term Human–Autonomy Teaming (HATs) to describe humans and intelligent, autonomous agents working interdependently toward a common goal (O’Neill et al., 2020; Lyons et al., 2021).

Human communication is naturally multimodal, with voice, facial expression and gestures considered as key channels supporting its realization. By analogy, these modes can be considered extremely relevant to achieve a similar natural exchange between the robot and the human teammates. On one hand, the robot must be endowed with the capability to not only accurately capture, analyze and understand the human states and requests, but also to reply adequately. On the other hand, the human teammate should provide information with a language that the robot can parse, and should be fully aware of the reactions and capabilities of the robot. Furthermore, in order to avoid the buildup of mistrust or frustration, he/she must be aware of any sudden change in the autonomy level of the robot and the associated behaviors.

One of the main contributions of this work is to elaborate on the generation of shared working plans relying upon a multi-modal human robot interaction and on empathy-relevant behaviors. In this interaction, the actors can have two types of roles: supervisor/control base and teammate. The human operator can assume both roles while the robot can assume only the latter one. As a supervisor, the human is responsible to define and adapt the ultimate goal of the mission as well as its main tasks. Furthermore, in this role the human also monitors and validates the team’s performance towards the team’s goals.

The teammate role implies that both human operator and robot execute actions and collaborate locally towards the completion of a mission through the multimodal interaction. The robot is autonomous in the realization of specific tasks by leveraging its capabilities to carry out MRO activities, but requires input from the human teammate and supervisor for the update of the goals and the planning of the mission. By design, the robot is not driven by intrinsic motivations and goals, and in this respect the human will be considered as the final authority responsible to specify the degree of automation expected for each task of the mission. However, the robot is capable of exhibiting empathy-like behaviors by leveraging its manufacturing capabilities to support the human teammate’s goal.

These capabilities have parallel representations as behavior tree leaf nodes across two main behavior subtrees of the HRI tree: the Safety HRI subtree and the MRO HRI subtree, as described in sections 3.1 and 3.2 respectively.

## 3 Framework

In the proposed framework we draw upon sophisticated levels of robotic perception and control to create an artificial sense of the environment and self-awareness, supporting the instantiation of intelligent robotic behaviors during the interaction with other entities in the environment. In particular, during the interaction with the humans the robot will firstly play the role of an observer, meant to assess the agent’s health status well before any further interaction (Section 3.1). If this preliminary screening identifies the human as capable of working, the robot proposes itself as a teammate for a sequence of scheduled MRO tasks (3.2).

Although far from being capable of displaying a full-blown empathic response, the robot offers an alternative to bridge the common *empathy gap* that characterizes social human interactions, i.e. the human tendency to underestimate the influence of varying mental states on our own behavior and to make decisions that only satisfy our current emotion, feeling, or state of being (Gutsell and Inzlicht, 2012). In this respect, we can argue that an autonomous robot can be an effective team player, which can exceed in certain situations the human capabilities. This is enabled by its greater information processing capability, the lack of performance fluctuations due to mood, personality or other psychological aspects, as well as no hindrance generated by human-like complications or daily struggles. In turn, a robot has a limited and very context-specific decision capacity, it cannot perform abstraction and undertake unexpected tasks, and lacks the dexterity of a human worker. These considerations can trigger a cost-benefits analysis of the empathy applied to HRI (Section 3.2.1), and a consequent estimation of risk levels for health, operations success and equipment condition (Section 3.2.2). With the support of its cognitive system, the robot evaluates each required operation and its capability of performing it (Section 3.2.3) and proposes itself to take over difficult tasks bearing high risks for human health (Section 3.2.4). This promotes a culture of safety in the workplace as the main benefit but without neglecting the cost associated with the risk transfer to the robot.

**FIGURE 2:**
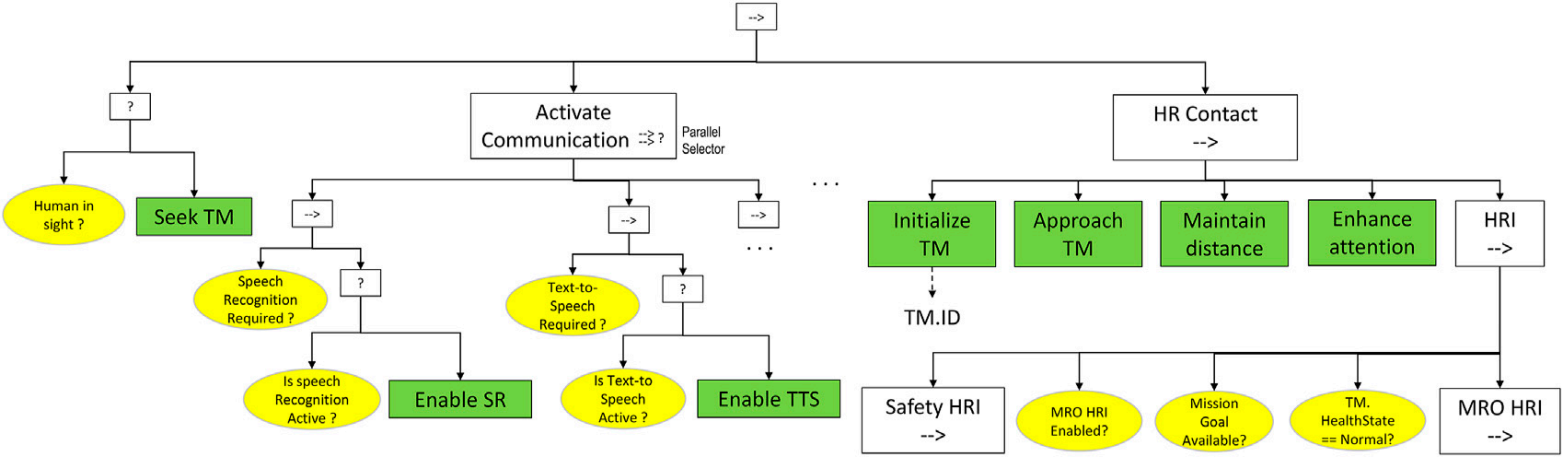
Root of the behavioral framework: human-robot contact and HRI sequence. The *HR Contact* subtree is executed only if a human teammate has been found and communication has been established. The *HR Contact* sequence initializes the data structure regarding the engaged teammate, approaches her/him, ensures safe and comfortable distance, and activates attention functions before starting the two main functions (Safety and MRO).

Starting from the premise the robot should support a *human teammate* (TM in the following) to carry out an MRO activity, the first behavior to be deployed by the robot is to establish the contact (Figure 2). This will be triggered by the satisfaction of the *human in sight* precondition. In case this returns Failure, the *Seek TM* action will be instantiated in order to localize the teammate. The detection of the human teammate triggers the activation of different communication channels (e.g., speech recognition, text to speech) followed by the implementation of a preliminary behavioral sequence.

This sequence, assuming the availability of autonomous navigation and the sensitivity to strategic affordances of interaction with humans, includes the following steps:

• Initialize TM: instantiates the teammate’s identity (ID property) and Position vector.
• Approach TM: assume a socially cognizant trajectory with adapted posture and slowing down as it nears the human.
• Maintain distance: the robot approaches the teammate always adhering to common conventions of what constitutes people personal space (e.g., social distance 3 m, personal distance 1.5 m, intimate distance 0.5 m).
• Enhance Attention: the robot’s operative system focuses resources on the sensors required to evaluate the health state of the human.

The last action of the *HR Contact* tree is to initiate the HRI sequence. The whole *HRI* behavior tree exploits information coming from the robot’s own sensors and from the control base by wireless connection. It includes the *Safety HRI* sequence followed by the check of a set of preconditions and finally the *MRO HRI* sequence, which will be described in detail in the subsequent sections.

### 3.1 Safety Subtree

Within our framework, the *safety* concept is addressed across different dimensions, which extend beyond the boundaries of the Safety BT. A first dimension is related to risks that have a physical impact on a person’s safety, such as collision risks. These risks are kept at bay through the execution of the *Approach TM* and *Maintain distance* sequence of activities under *HR Contact* BT. Secondly, the consideration of psychosocial measurements within the *Safety HRI* subtree contributes to the in-the-moment assessment of the health state and cognitive overload of the human teammate. It is assumed that if the robot is perceived to be dangerous, then the TM affective state would be tense. This can be addressed by the robot with an adapted questionnaire during the verbal interaction with the TM (i.e. *Safety HRI/Interaction* subtree). Overall, the robot promotes a safer workspace by indicating or preempting a danger either for the human health, for the success of a task or for the plant functioning and formulating a proper reaction to them (i.e., *Safety HRI/Deliberation* subtree). Furthermore, the integration of the human behavioral factors into the planning of the human-robot shared activities aims to safeguard the human health status by assigning high-risk tasks to the robot (i.e. *MRO HRI subtree*) if potential dangers are detected in the current plan.

**FIGURE 3:**
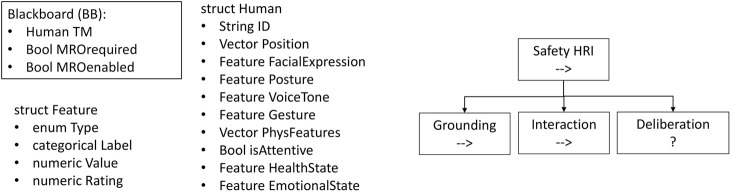
Phases of *Safety HRI* and related blackboard variables. The blackboard variables allow to store human teammate identity and health/emotional state, supporting the execution of the Safety HRI sequence.

In particular, the Safety BT contains the conditions and actions carried out by the robot to assess the health state of the actively engaged human teammate and subsequently to deliberate on alternative courses of action. The Safety HRI sequence consists of three subtrees (Figure 3):

• *Grounding*: determines the teammate’s health state by processing information acquired by sensors.
• *Interaction*: tries to obtain further information by explicit verbal interaction with the teammate.
• *Deliberation*: takes an action consequent to the determined HealthState of the teammate.

Moreover, the tree’s blackboard includes the following objects:

• The data structure TM (Teammate) of type Human, which collects information about the human worker presently involved in the human-robot team.
• The MROrequired Boolean variable, which enables the execution of the MRO HRI subtree, based on the mission goal.
• The MROenabled Boolean variable, which is turned off if the conditions for proceeding to the MRO HRI after the Safety HRI are not satisfied.

#### 3.1.1 Grounding (Sequence Node)

**FIGURE 4:**
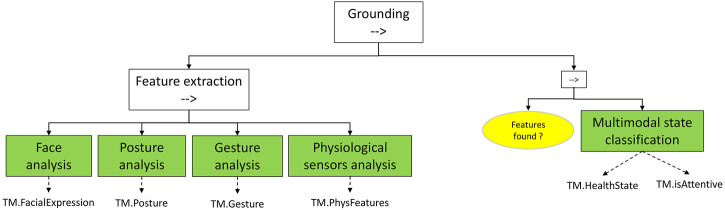
*Grounding* subtree. *Feature extraction* uses multiple signals (e.g., vision, ECG) to generate inputs for the *Multimodal state classification* model.

The *Grounding* sequence (Figure 4) is composed by two subtrees:

• Feature extraction: uses all the available sensors to extract features related to health state expression. The following options exemplify commonly analyzed aspects, but others can be included within the same framework:

o Facial expression (happy, focused, nervous, sad, inexpressive, eyes open/closed).
o Posture (lying, standing, sitting, squatting, hanging).
o Gesture (pointing, typing, standing relaxed, walking, climbing).
o Physiological signals (heartbeat rate, EEG spectrum, other features computed from time series signals).

In this case, the action nodes that execute feature extractions return success at the end of the operation, both in case of successful and unsuccessful feature recognition. The availability of features to analyze is managed by the following sequence:

• Multimodal state classification: if at least one feature has been found in the feature extraction phase (the check is managed by the *Features found?* condition), the health state classifier uses all the available ones to determine two labels: the *HealthState* of the teammate and whether he/she is paying attention (*isAttentive* variable) to the robot or not.

#### 3.1.2 Interaction (Sequence Node)

**FIGURE 5:**
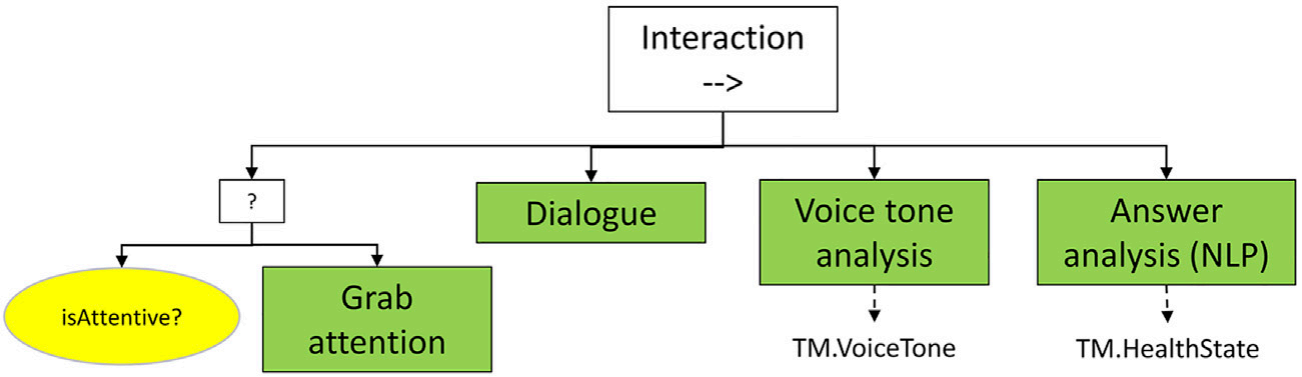
*Interaction* subtree. After ensuring the human’s attention, a dialogue sequence allows to enrich the emotional state by voice tone, and to obtain direct information from verbal communication.

After the first phase of *HealthState* determination, the robot tries to collect further information by directly communicating with the teammate (Figure 5). This is achieved by the following sequence:

• *Grab attention*: is the teammate attentive (*isAttentive?* condition), the robot tries to obtain her/his attention, for example by calling her/him by TM. ID.
• *Dialogue*: a series of questions and answer collection phases aims at narrowing the *HealthState* uncertainty by direct comments (e.g., “How are you?” followed by an acceptable answer by the human teammate).
• *VoiceTone analysis*: another pre-trained classifier, focused on voice tone recognition, enriches the multimodal information.
• *Answer analysis*: the answers received in the *Dialogue* phase are parsed by Natural Language Processing methods, potentially modifying or adjusting the provisional *HealthState.*

#### 3.1.3 Deliberation (Selector Node)

**FIGURE 6:**
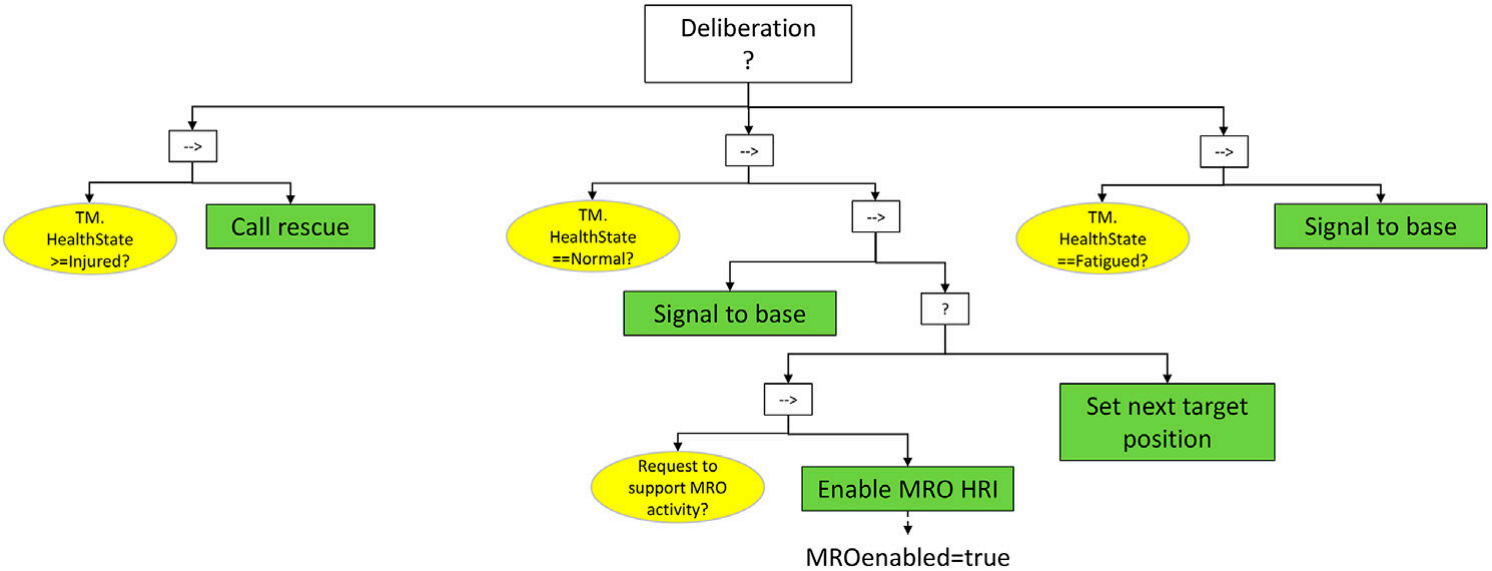
*Deliberation* subtree. The determined health state is used to decide whether the teammate needs urgent medical assistance or if it can and must be engaged for a collaborative MRO activity.

The Deliberation node (Figure 6) evaluates the teammate’s *HealthState*, and determines the action to take. The proposed criterion is a proof of concept, easily customizable to the policies that the employer implements in the target plant. In this example, three cases are managed (notice that the evaluation is managed by a selector between three sequences):

• T*M.HealthState≥Injured*: if the *HealthState* corresponds to *Injured* or a more severe estimated condition, rescue is directly called for the human worker in danger.
• T*M.HealthState == Fatigued*: if the is able to proceed but her/his state is evaluated as fatigued, the situation is signaled to the control base, and the robot asks directly to the teammate if he/she feels that the manufacturing phase (if required) can be undertaken.
• *TM.HealthState == Normal*: if the worker is healthy, the clearance is signaled to base and if there is a request to support MRO activities then the variable *MROenabled* is set to true. In case no request for such activity is needed the robot can, for instance, sets the target position towards which to navigate next and the *Safety HRI* subtree can be exited with a Success status.

### 3.2 MRO HRI Subtree

**FIGURE 7:**
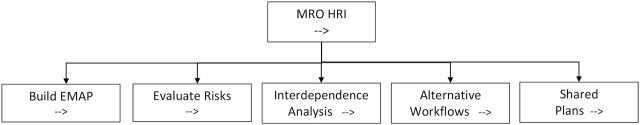
Main structure of *MRO HRI* tree.

The *MRO HRI* subtree, whose main phases are illustrated in Figure 7, contains the conditions and actions required to address several key decisional issues that support the planning of shared MRO tasks with the human teammate. It is executed next to the *Safety HRI* subtree only if a number of pre-conditions evaluate to true (see Figure 2).

The *MRO HRI* subtree has been designed with the aim of achieving the following objectives:

- Relieving operators from executing tasks associated to high risks for health;
- Relieving operators from stressful working conditions and promoting their involvement in high added value tasks;
- Reducing the complexity of on-site preparatory and post-intervention activities, whenever the robot requires less preparatory measures before and after performing a MRO operation;

Recent studies emphasize how mental state of the workers and job-related risk factors are affecting productivity (Di Maria et al., 2020) and safety (Choi et al., 2019) in the workplace. For this reason, the proposed approach to shared human-robot task planning aims to include also aspects of human affection, like emotional dispositions and sensitivity. This builds upon affective grounding concept (Jung 2017), i.e., the sequential evaluation of the health and emotional state of the human teammate while grasping also his/her intention with respect to the MRO activities to be carried out. For instance, the prior execution of the Safety subtree may return a *Normal* health state for a human teammate, who nonetheless exhibits a stressed attitude, sensed from his/her voice. This will trigger further investigations into possible emotional barriers that might hinder the satisfactory completion of the planned MRO tasks.

The psychosocial measurements made available through the Safety HRI are an essential starting basis not only to enhance the human safety but also to foster the empathic capabilities of the robot. Empathic capabilities are triggered by human behavioral cues and have a compelling influence on the outcome of the deliberative process of the robot. Generally, these capabilities can be analyzed under two main categories, the ones that are more emotional and the ones that are more cognitive. Emotional empathy can be defined as the ability to experience and understand another’s person affective experience by sharing the same feelings, whereas the cognitive empathy refers to the ability to represent and understand the internal mental states of someone and to be able of perspective taking.

In our framework, the empathy concept is limited. The definition of feelings does not need to be comprehensive for what concerns the human experience: it just needs to be a collection of worker conditions that are relevant to our reference setting. The capability to attribute mental states is linked with robot’s relevance to adapt its behavior in order to accommodate teammate’s interaction involvement. In this respect, the robot’s actions deployed across both *Safety HRI* and *MRO HRI* trees promote a shift from thinking about empathy as an emotional process to thinking about empathy as a behavioral decision system. The focus of this system is on a dynamic, iterative process of robot’s goal prioritization to improve the occupational health and welfare and to relieve the human teammates from unpleasantly feeling or difficult tasks.

To account for the emotional and physical involvement, as well as the sensory perceptions of the human teammate in relation to contextual conditions, we propose the use of an *empathy map*, as described in the following section.

#### 3.2.1 Build Empathy Map

The empathy map is used as a representation of the MRO personnel’s mindset, actions and risks in relation with specific use case scenarios and associated tasks. Such data structure allows to collect valuable insight for the practical implementation of a shared human-robot work plan.

**FIGURE 8:**
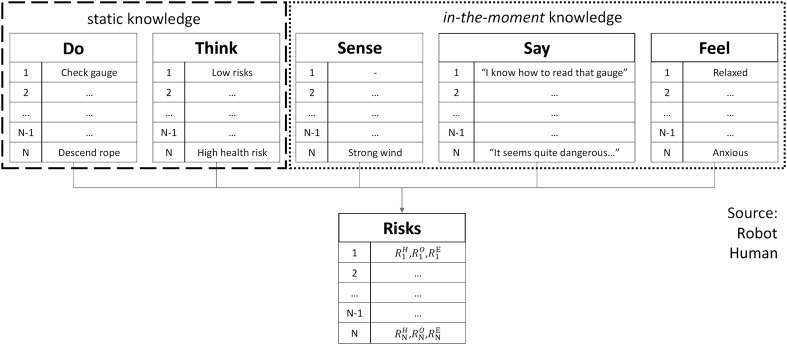
Empathy map. “Static knowledge” dimensions (*Do* and *Think*) regard conditions known before considering contextual factors. “In-the-moment knowledge” dimensions (*Sense*, *Say* and *Think*) regard factors that depend on the present state of the environment and of the involved human teammate.

Empathy maps are tools that are neither chronological nor sequential (Campese et al., 2019). Nevertheless, we propose a slightly different interpretation to the definition of the conventional dimensions, by introducing a task sequential index (from 1 to N) and by including in-the-moment emotionality. The proposed empathy map is therefore composed by the following dimensions, represented and exemplified in Figure 8 **:**

• **Do**—encloses the sequence of actions the human operator is supposed to execute in order to complete a MRO activity. The sequence may be communicated directly by the teammate during the current human-robot interaction or made available previously by the control base; the latter case will be considered, for simplicity.
• **Think**—provides risk baselines based on aggregated heuristics learned from MRO personnel, using historical data (e.g., incident statistics, operator interviews, qualitative research) and capturing the experiences and opinions of workers in terms of feelings and risks (human health, process, equipment associated with the tasks).
• **Sense**—information collected on-the-job by the robot, to enrich the active context (e.g., measurements from anemometer, thermometer, vision, GPS, weather forecasts).
• **Say**—collects contextual information provided by the human teammate during the actual human-robot interaction (e.g., *“task T2 is very long and requires additional equipment and human resources”*).
• **Feel**—captures the human teammate feelings about the job at hand during the actual human-robot interaction (e.g., overwhelmed, worried, stressed, calm). The emotional measures can be based on the circumplex model of emotions, which is a widely used bidimensional model used to capture a wide range of positive and negative affect encountered in common HRI scenarios (McColl et al., 2016; Cavallo et al., 2018). According to this model, the human emotional states represent different levels of intensity (*arousal*) and pleasantness/unpleasantness (*valence*). The focus can be directed towards fundamental emotions like *happy, stressed, relaxed, sad, alert* prior to the definition of the shared plan.

These five dimensions contribute, for each task i=1,…,N , to the determination of risk values in three categories, which we have identified as the most relevant for industrial contexts that could benefit from the support of human-aware mobile robots:

• *Health Risk* $R_{\text{i}}^{H}$ : represents the risk for the safety of the human worker e.g., of being injured or getting in contact with hazardous materials.
• *Operational Risk* $R_{\text{i}}^{O}$ : represents the risk of compromising the quality or the success of the task in progress, e.g., performing an invalid defect inspection scan, performing a faulty repair operation or compromising work schedule.
• *Equipment Risk* $R_{\text{i}}^{\text{E}}$ : represents the risk of damaging assets or equipment, including the robot itself.

#### 3.2.2 Risk Assessment

Demanding cognitive and physical tasks are more prone to error-producing conditions, and an inadequate psycho-physical state of the human could increase the likelihood of risk modes. The aim of the proposed framework is then to mitigate these risks thanks to the support of human-aware robotics.

Before engaging into direct interaction with the human teammate, the robot plays the role of an external observer. The starting point is the identifications of possible *human errors* and *at-risk behaviors*, which could possibly lead to accidents, or affect reliability, productivity and efficiency of operations and equipment.

For what concerns the errors, they can be classified in two types: *endogenous* and *exogenous*. Endogenous errors are defined as caused by negative personal performance shaping factors, such as anxiety, stress, fatigue, fear, sensory processing disorders and other psychosocial factors. Performance in the workplace is also challenged by the role of the endogenous circadian rhythm (Pilcher and Morris, 2020). Exogenous errors, on the opposite side, are caused by negative environmental performance shaping factors, such as low light conditions, fatigue and distraction patterns, technology glitches, weather condition, the absence of adequate tools and equipment, and others.

*At-risk behaviors* are different from *human errors*. They can be described as behavioral choices that are taken when the perception about the risk of a task is mistakenly interpreted as insignificant or justified. The incapability to see the risk in these situations might have different causes, such as the development of unsafe habits or a natural drift from compliance with specific working procedures. These factors are accentuated by the accumulation of experience in realizing a specific job, which promotes tolerance towards at-risks behaviors and ultimately induces failures in recognizing risky situations.

Quantitative risk evaluation is an essential step to enable automated decision making about the formulation of improved human-robot shared plans. In particular, calculated risk values can be used to select candidate sub-tasks for being supported or completely taken over by the robot teammate. To this aim, we suggest an approach that is compliant with the standard definition of *risk* given in International Organization for Standardization, (2018), where risk associated to a task is characterized by a *consequence* value C and a *likelihood* value L . Consequence quantifies the seriousness of the potential accident and can be defined on an arbitrary severity scale, e.g. ranging from 0 (no consequence) to 5 (highest severity), while likelihood indicates the “chance” that the accident occurs. The commonly used formula for taking into account these two measures quantitatively in a global risk assessment is

R=C⋅L.

Aust and Pons (2021) suggest an extended framework for risk assessment that involves the use of *cofactors*, which are risk modifiers based on situational conditions, like weather, human health and affective status, equipment condition, etc. In particular, they propose the use of multiplicative cofactors $x_{j}$ , j=1,…,Q , with risk taking the form

$$R=C\cdot L\cdot \prod_{j=1}^{M}x_{j}.$$

This formulation is well suited for the approach proposed in this work: in particular, the baselines of consequence and likelihood for each subtask can be defined within the *Think* dimension data structure, while the *Sense*, *Say* and *Feel* dimensions define the situational cofactors that modify the total risk. However, instantiation of such cofactor values from historical data and expert opinions is difficult, since likelihood is defined on an arbitrary, subjective scale, which in (Aust and Pons, 2021) is defined from 1 to 5, while cofactors are scaling factors ranging from 0.5 (halved risk) to 2 (doubled risk). For this reason, we propose a slight modification to this approach, adopting the natural mathematical interpretation of likelihood L as a probability. To stress this aspect, we will denote now likelihood as p . In this way, risk R can be interpreted as the expectation of consequence on a large number of executions of the same task. For example, referring to the health risk category, a value of 0 may be achieved only when the potential for injury is impossible, while a value of 0.4 can be reached if the severity of an occurred accident is 4 out of 5 (e.g. serious physical injury) and the frequency of the accident, based on historical data, is p=0.1 . Notice that risk must be evaluated on a different scale with respect to consequence, although they both have equal minimum and maximum values: in the aforementioned example, a 10% likelihood of class 4 consequence could be considered unacceptable, even though the calculated risk is 0.4.

In this framework, instead of using each cofactor as a multiplier to the baseline risk, we associate an additive likelihood offset $\Delta p_{\text{j}}$ to each possible cofactor $j=1,\;\ldots M_{i}$ , where $M_{i}$ is the number of relevant cofactors for the i -th task. This formulation simplifies the implementation of cofactors from historical data of task outcomes, by tabulating accident frequencies for each previously observed combination of cofactors in field operations; moreover, cofactors that can be considered task-independent can be estimated from accident historical data, since their effect on total risk does not depend on the value of likelihood itself, as in the multiplicative case. Therefore, the three global risks for a subtask i can be formulated as follows:

$$R_{i}^{H}=C_{\text{i}}^{\text{H}}\cdot \left(p_{\text{i}}^{\text{H}}+\sum_{j=1}^{M_{i}}\Delta p_{\text{j}}^{\text{H}}\right),$$

$$R_{i}^{O}=C_{\text{i}}^{O}\cdot \left(p_{\text{i}}^{O}+\sum_{j=1}^{M_{i}}\Delta p_{\text{j}}^{\text{O}}\right),$$

$$R_{i}^{E}=C_{\text{i}}^{\text{E}}\cdot \left(p_{\text{i}}^{\text{E}}+\sum_{j=1}^{M_{i}}\Delta p_{\text{j}}^{\text{E}}\right),$$

where apices H , O and E identify the terms related to Health, Operational and Equipment Risk respectively. If the decomposition of a task in subtasks is available, its total risk severity can be estimated as the maximum value across all associated subtasks.

**FIGURE 9:**
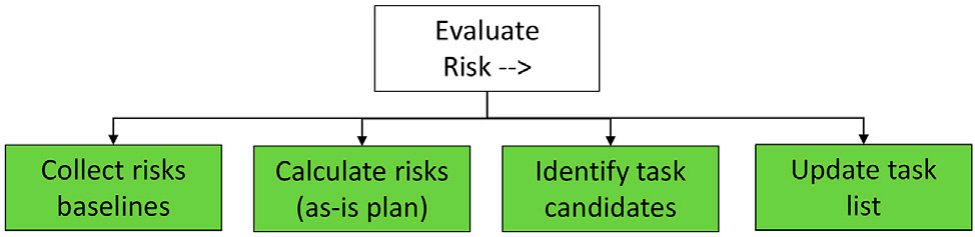
*Evaluate Risk* subtree. The sequence uses known risk baselines to calculate a risk value for each task, so to identify tasks that could be assigned to the robot in a revised task list.

Specific thresholds will be imposed for each risk category, with a particular focus on guaranteeing low health risk $R_{i}^{H}$ for each task i . In either case, a task with unacceptable risk value will be flagged as a candidate for potential execution by the robot, following the sequence described in Figure 9. The execution of the *Evaluate Risk* sequence aims to assess the risk for each task within the as-is task plan and to identify the potential candidates for being taken-over by the robot.

For a better understanding of the framework, relevant activities are selectively exemplified in the subsequent sections considering as use case the *in-situ* inspection, maintenance and repairing of the leading edge of a wind turbine blade. The capabilities of the robotic platform considered for this use case fit the description of section 2.2.3.

*Example 1. Initially, the robot is transported by car to the wind farm location; after its start routine and the execution of the Safety HRI subtree it evaluates the health state of the designated teammate as normal. Next, through the execution of the Build EMAP subtree, the robot engages into a dialogue with the teammate, during which it receives the mission information (goals and list of tasks). In particular, subtask k, reaching a damaged blade by climbing down a rope, has consequence C = 3 (the worker oscillates on the rope and gets injured by hitting it) with likelihood *p* = 0.05 (i.e., the frequency of such accidents in normal conditions). Moreover, it senses a form of hesitation in the teammate’s voice with respect to the execution of the blade inspection via rope access, due to the windy weather $\left(\Delta p_{1}^{\text{H}}=0.1\right)$ . The robot activates its anemometer and detects a wind speed exceeding 10 m/s $\left(\Delta p_{1}^{\text{H}}=0.05\right)$ . The total risk level for health related to this subtask is then $R_{k}^{H}=C_{\text{k}}^{\text{H}}\cdot \left(p_{\text{k}}^{\text{H}}+\Delta p_{1}^{\text{H}}+\Delta p_{2}^{\text{H}}\right)=0.6$ . Upon the completion of the Evaluate Risk subtree for all tasks, the inspection and repairing of the blade are identified as candidates for take-over, on the grounds that they are characterized by high health risk levels due to exogenous factors (working-at-heights, adverse weather conditions and exposure to dangerous chemicals, e.g., epoxy, paints)*.

Some additional examples are proposed in Table 3, where risk quantification is detailed considering two cofactors. The next step consists in verifying the robot’s fitness for the updated task list, i.e., checking if the capabilities of the robot match the task requirements.

**TABLE 3:** Risk computation for different events within the wind turbine blade example. Type labels H, O, E represent Health, Operational and Equipment risks respectively. C and p are consequence and likelihood. CFk stands for “cofactor number k”.

Event	Type	C	p	CF1	CF1 Value	CF2	CF2 Value	Risk
Swing on rope - injury	H	3	0.05	Nervous operator	0.1	Strong wind	0.05	0.6
Swing on rope - tool crash	O	5	0.1	Nervous operator	0.1	Strong wind	0.05	1.25
Swing on rope - tool crash	E	4	0.1	Nervous operator	0.1	Strong wind	0.05	1
Swing on rope - injury	H	2	0.03	Nervous operator	0.1	Strong wind	0.05	0.36
Swing on rope - tool crash	O	5	0.1	Nervous operator	0.1	Strong wind	0.05	1.25
Swing on rope - tool crash	E	4	0.1	Nervous operator	0.1	Strong wind	0.05	1
Self-injury with tool	H	4	0.1	Nervous operator	0.03	Unexperienced operator	0.2	1.32
Brush wearing	O	1	0.3	Unexperienced operator	0.4	Tool malfunction	0.01	0.71
Brush wearing	E	1	0.3	Unexperienced operator	0.4	Tool malfunction	0.01	0.71

#### 3.2.3 Interdependence Analysis

**FIGURE 10:**
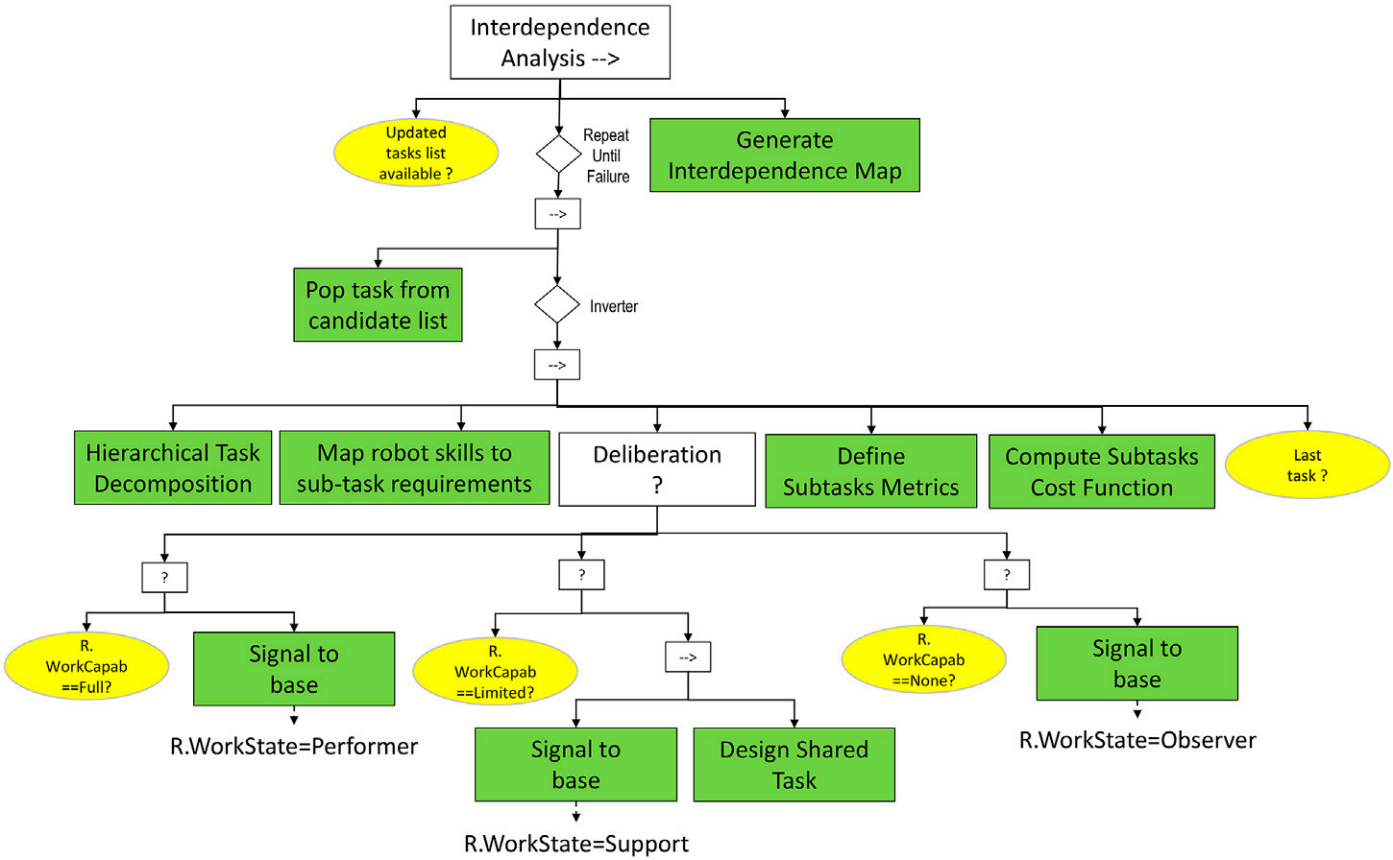
*Interdependence Analysis* subtree. The *Repeat Until Failure* decorator node allows to scan each task from the candidate task list. For each subtask, the robot’s capabilities for performing it are considered (*Map robot skills to subtasks* requirements node), and a task-dependent metric is used to assign a score to each possible subtask assignment.

Humans and automated systems have been traditionally assigned to separate functions, but recent advances permit a more fluid relationship approximating human-robot teaming paradigms (Lyons et al., 2021). Ideally, a well-functioning team relying upon the human-robot interaction to solve problems in real industrial settings needs an appropriate approach to enable the synchronization of their skills and capabilities. Generally, human-human and human-robot teaming comes in many forms with different characteristics, but the most fundamental concept consistently addressed in literature is interdependence (Johnson et al., 2018). In our formulation, the interdependence analysis, as described in Figure 10, can be extended beyond pure task dependency, and is aimed at designing workflows consisting of a mix of joint and independent activities.

The tasks considered for a different human-robot workload distribution are mainly the potential candidates defined in the *Evaluate Risk* sequence, i.e. the ones with a high intrinsic health risk. The *Interdependence Analysis* relies upon a child sequence subtree designed to iteratively assess the robot’s ability to perform for each task from the candidate list. Firstly, a hierarchical decomposition based on the human-aware task planning formalism (Lallement et al., 2014) is carried out, aiming to structure a high-level task representing a manufacturing goal into subtasks which must be executed by the members of the HAT. Secondly, a mapping between the requirements of each subtask and the capabilities of the robot is realized. Following upon the outcomes of the mapping activity, a decision is made regarding the robot’s fitness for the job, considering three possible scenarios: 1) the robot has full work capability to solve the task (i.e., the working state of the robot is updated to *Performer* role), 2) the robot needs support to solve the task (i.e., the working state of the robot is updated to *Support* role) and 3) the robot cannot provide any form of assistance to solve the task (i.e., the working state of the robot is updated to *Observer* role). By default, a task considered critical for the human health can be fully subsumed by the robot only if the required capabilities are available, i.e., the robot has the sensing, locomotion and working skills required to fully perform the task reliably. In other cases, the human assistance might be required due to either a lack of robot capability or to simply enhance the reliability of the process. In this respect, the *Design Shared Task* activity aims to identify a suitable distribution of the subtasks of any shared human-robot task by defining the structure, process and nature of the potential interactions between them.

*Example 2. Before starting any inspection or repair operations, the robot needs teammate assistance to reach the blade surface. The conventional setup approach used by human workers, containing the subtasks ST1-Climb inside the windmill tower, ST2-Access the nacelle, ST3-Lock the rotor and ST4-Hang the rope, cannot be executed autonomously by the robot. This setup task is redesigned for a human-robot interaction, considering two additional subtasks: ST5-Hoist the robot (teammate) and ST6-Attach robot to the blade (robot and teammate). For the last subtask, the teammate operating the rope attached to the robot gets access to the images transmitted by the robot’s camera, to facilitate its precise positioning on the blade (the teammate is the performer of the subtask, while the robot assumes the support role)*.

If the robot is unable to provide any form of support towards the completion of a task, it can assume an *Observer* role, to enable a *learning from demonstration* process based on the observable human’s routines and working environment.

The completion of a subtask can involve a particular sequence of actions which promote interactions between the teammates at various levels of abstraction. At this stage, additional metrics suitable for the manufacturing environments need to be defined. These metrics are supposed to complement the risk values calculated previously for each task, to support the definition of appropriate cost functions for a holistic assessment of the possible task plan alternatives and eventually for the selection of the best fit plan for a specific scenario. While in the manufacturing field time efficiency and quality are crucial expectations, other metrics capable of giving an indication of the human wellbeing based on ergonomic considerations are recently gaining momentum (Busch et al., 2018; Gualtieri et al., 2020; Cardoso et al., 2021).

Once all tasks from the candidate list are processed, an *Interdependence Map* will be generated, reporting the role of each teammate together with the adequate timeline, context-dependent cost function and associated risk. This centralizes the role of each teammate, the capability to either directly perform the work or to ensure a supportive function, and the nature of interdependences, with indication of possible limitations and reliability levels across all tasks.

#### 3.2.4 Alternative Workflows

**FIGURE 11:**
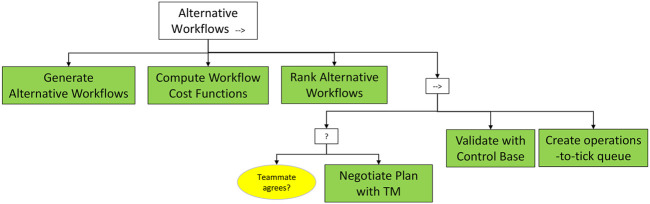
*Alternative Workflows* subtree. Alternative subtask assignments are proposed to the human teammate, based on their total cost, and agreed with the teammate and with the Control Base.

The *Alternative Workflows* sequence (Figure 11) aims at defining the final Shared Plan to be executed, based on the *Interdependence Map*. The first step is the generation of multiple alternative workflows to accomplish the mission goal. Each workflow gives full consideration to the emotional status, preferences and capabilities of the human teammate, as well as to the robot’s capabilities to encode the sequence of actions necessary for task completion. The degree of optimality of each generated workflow is computed by a cost function, tailored to the end user and the specific task, which considers not only the risk dimensions but also the selected metrics across the entire sequence of tasks. The total costs of each workflow is employed to generate a ranking of the alternatives, based on which they will be proposed to the human teammate.

The plan validation is deployed with the support of a negotiation-based platform enabling multi-turn, bidirectional communication between the robot and the teammate, which allows the human to review each plan proposed by the robot and to either accept it or ask for a different proposal. At the end of the process, the robot will establish a direct communication channel with the control base and will leverage its feedback as the ultimate decision authority to validate the plan by overriding any other teammate.

The last activity within the *Alternative Workflows* tree consists of the generation of a list containing the complete execution sequence of the constituent tasks of the shared plan (i.e., *operations-to-tick* queue).

*Example 3. As a result of the successful completion of the Interdependence Analysis and Alternative Workflow subtrees, the operation-to-tick queue for the automated leading-edge repair of the turbine consists of the following sequence of behaviors: BT1-Setup (as previously described in Example2), BT2-Inspection (detailed blade inspection before repair), BT3-MRO Prep (surface preparatory operations before repair), BT4-Setup (descend to the ground, unload the cleaning tools and load the repairing tools, hoist back to the repairing area on the blade surface), BT5-MRO Repair (actual repairing of the damage), BT6-Inspection (detailed blade inspection after repair), BT7-Setup (descend to the ground once the repairing is complete). The robot and the human teammate are deploying either individual or collaborative behaviors to complete the mission.*

#### 3.2.5 MRO Shared Plan

**FIGURE 12:**
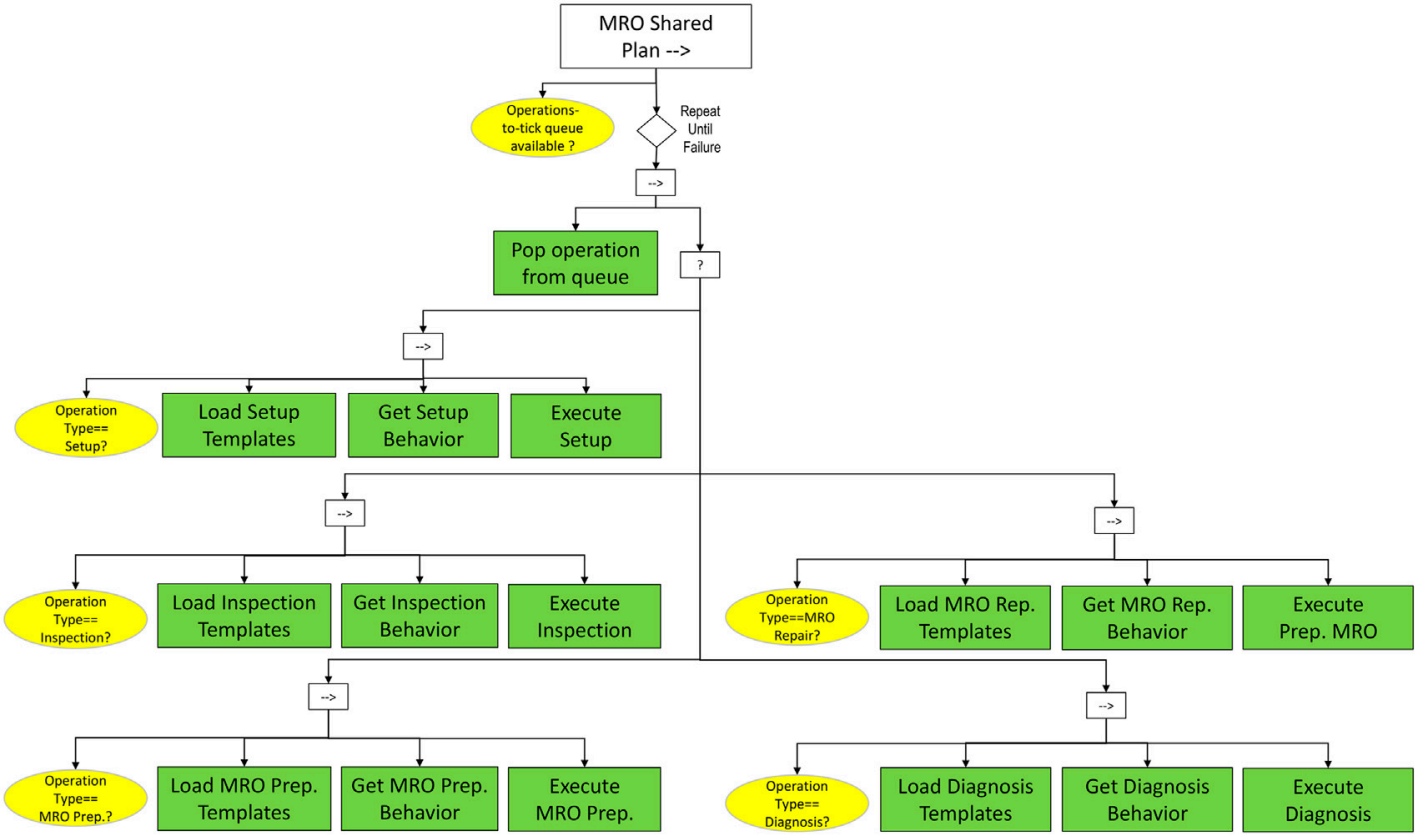
*MRO Shared Plan* subtree. The subtask queue is processed to perform each scheduled operation. In this framework, the possible operation templates are Setup, Inspection, MRO Preparation, MRO Repairing and Diagnosis.

In contrast to the *Alternative Workflows* subtree, the *MRO Shared Plan* subtree, depicted in Figure 12, is not only responsible for decision-making but also for monitoring, execution and adaptation of behaviors to carry out MRO activities. This relies on the development of behavior libraries consisting of reusable, parametric behavior trees, which selectively deploy adapted operational behaviors to cope with diverse manufacturing scenarios.

Starting from the root (*MRO Shared Plan* sequence), the *operations-to-tick queue* is loaded from the blackboard and with the support of a repeater node the corresponding operations are initialized and executed in the given order. Further down, a selector defines which behavior subtree needs to be expanded and instantiated for execution. The tree exploits logical pre- and post-conditions as well as sensory data to monitor the execution of each behavior.

Five behavior types are currently exposed in the aforementioned tree, namely setup, inspection, diagnosis, preparatory MRO and repair MRO. Some of them may have precedence constraints, assumed to be linked with the instantiation of re-usable behaviors that the robot carries out to complete the overall goal of an operation, if adequate conditions are met. The reuse of a behavior is enabled by a behavior reference task. This reference task enables the running of another behavior tree within the current behavior tree aiming to overcome limitations of simple planning scenarios and to extend the behavior of the robot to accommodate the deployment of various scenarios. Upon the reload of an external behavior tree a new instance of the operation data structure is created considering the elements provided by the associated operation template.

*Example 4. As part of its initial blade mission, the robot needs to deploy twice the detailed inspection behavior (as described in Example3): 1) the first inspection (BT2) consists of a laser scanning, used to generate a 3D model to assess the blade condition and to adjust the tools required before the repair operation; 2) the second inspection (BT6) consists of a laser scan of the blade after the repair, to validate the results and to guarantee the highest quality. Both inspection behaviors employ the same BT structure but with differently parametrized templates based on teammate’s input.*

**FIGURE 13:**
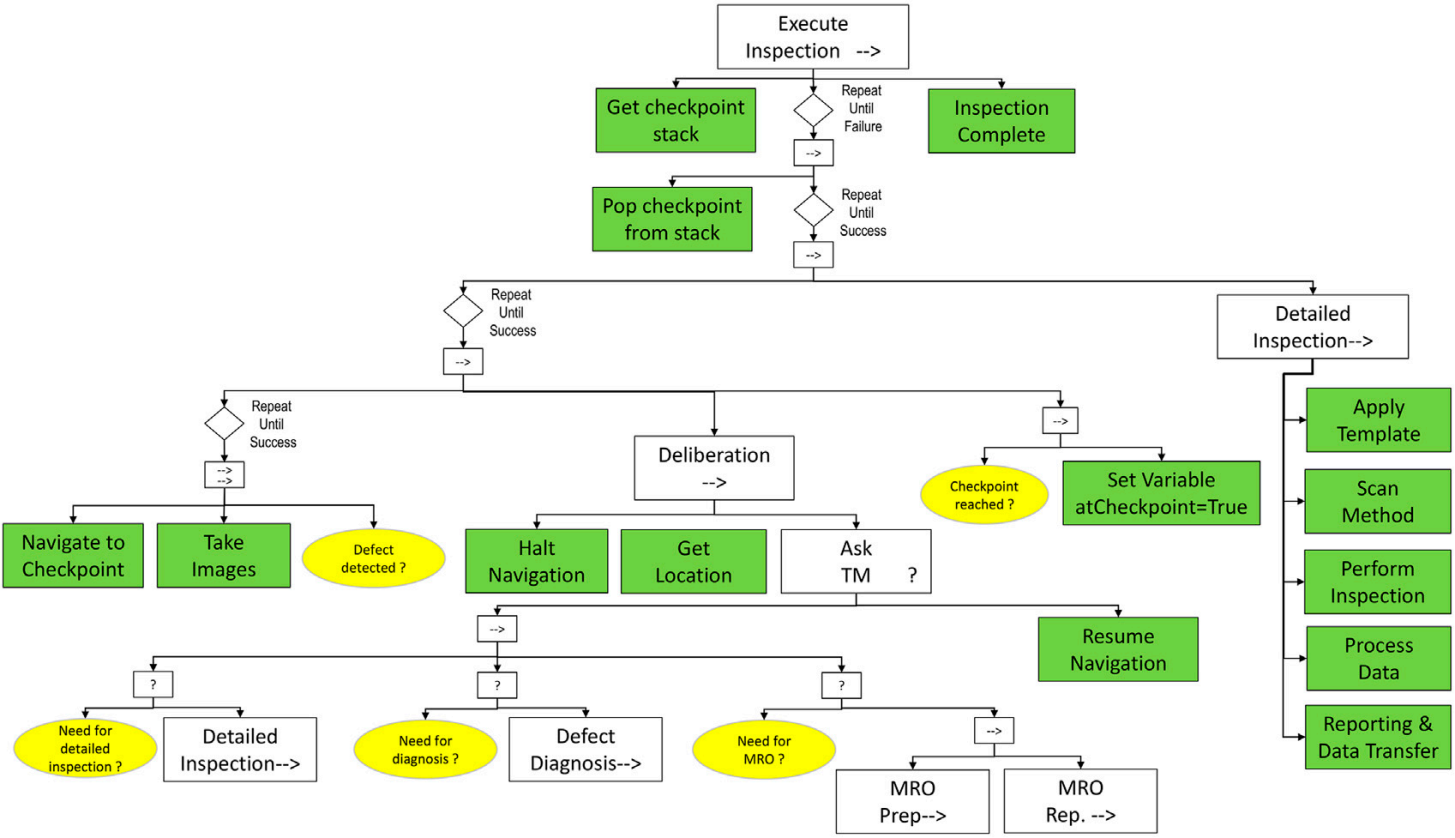
*Inspection* subtree. Inspection checkpoints are provided in a stack, from which they are extracted for processing. For each checkpoint, the robot navigates to its coordinates to capture images (leftmost subtree). When a defect is detected, the local *Deliberation* subtree is activated, and the human teammate is requested to provide further instruction on what to do (*Detailed Inspection*, *Defect Diagnosis*, *MRO* Operation). If a Detailed Inspection is needed, it is executed based on a sequence of actions designed ad-hoc for the used measurement system.

A possible configuration of an *Execute Inspection* subtree is proposed in Figure 13.

We define the *inspection* behavior with the support of robot’s capabilities to navigate to a specific location and to deploy an inspection method that can function independently of lighting conditions, contrast, reflection and position. A list of checkpoints to be visited consecutively by the robot can be provided initially by the teammate. As long as the next checkpoint is not reached, the behavior of the robot is driven by a parallel sequence controlling the navigation along the predefined trajectory, while continuously taking images of the surface and checking for defects. If there are any signs of surface defects, a *Deliberation* sequence is triggered, since the robot needs to decide to either carry out further investigations or to continue its navigation. In the former case, an adapted form of communication with the teammate will accompany the unfolding plan, leading to the translation of her/his feedback into a sequence of additional tasks to be performed in place (e.g., repairing of the defect after a prior detailed inspection, diagnosis and surface preparation). This sequence will fail if at least one of the child conditions is not satisfied, i.e., if no further operation is required by the human at the present position; in this case, the robot will resume the navigation towards the next checkpoint. As soon as this is reached, the planned inspection operation can take place. The robot employs adequate operation templates selected from a predefined library based on the outcomes of the previous phases of the *MRO HRI* subtree, including human teammate’s feedback.

Practically, an inspection template collects the minimum set of requirements needed to organize, plan and keep track of the entire sequence of activities to implement the inspection operation (e.g., which type of inspection systems/methods needs to be used? which type of defects are supposed to be found on the inspected surface? how to setup the operation to get the best outcomes?). These will be followed by data processing (e.g., cloud to mesh), and a documentation activity addressing the reporting and transfer of the acquired data to the control base.

The priority shift from a sequence of detailed inspections to an unplanned task is handled by the *Deliberation* node and can be implemented exclusively after a dialogue with the teammate, i.e. execute the *Ask TM* behavior.

*Example 5. While navigating towards the next inspection checkpoint, the robot detects the presence of a damage on the blade surface. It stops and establishes a communication channel with the human teammate, which confirms the need to perform a detailed inspection and diagnosis. Next to inspection, the diagnosis task is deployed across the following behaviors: BT1-Identify damage scenario (robot): leading edge erosion through laminate; BT2-Assess damage criticality (robot): critical damage, an immediate action is required to prevent turbine failure; BT3-Deliberation (robot): the confidence level with respect to the damage assessment is not high, therefore the robot ask human support; BT4-Validation: the teammate analyzes the robot’s report and recommends immediate repairing of the damage (after evaluating the damage category, its location, the impact and time required for repairing activities), to ensure safe turbine operation; at the end of this subtree, the robot sets the variable MROenabled to true (teammate and robot). This validation outcome puts on hold the current navigation towards the next target checkpoint until the damage situation is cleared by the MRO behavior of the robot.*

**FIGURE 14:**
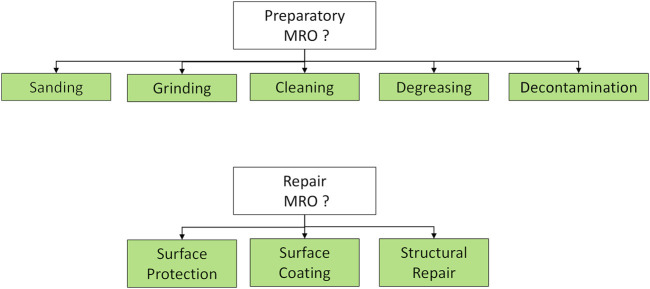
Examples of *Preparatory* and *Repair MRO* subtree selectors. In each case, the proper preparation or repairing technology is selected among the ones available to the specific robot.

The diagnosis supports also the selection of the appropriate MRO scenario considering both preparatory and repairing operations (Figure 14).

*Example 6. In order to prepare the damaged surface for repair, the robot instantiates and executes sequentially three preparatory MRO behaviors: BT1-Sanding to remove the damaged layers (sander tool); BT2-Cleaning to remove any remaining dust from the sanding (brush tool); BT3- Degreasing to remove all surface contaminants (spray tool). After each preparatory step, a tool change is required (realized by the robotic arm installed on the platform).*

*Example 7. Based on the diagnosis outcome and teammate’s feedback, the robot selects the surface coating behavior to carry out the repair of the leading edge of the blade. The following subtrees will be sequentially deployed: BT1-Sense environmental conditions on the blade surface (robot): wind, temperature and humidity; BT2-Ask teammate (robot and teammate): the robot provides a report on the environmental conditions and checks the status of the repairing material and tool, while the human uploads the correct process recipe (robot and teammate); BT3-Filler application (robot): apply the exact amount of filler material; BT4-Profiling (robot): create a smooth finish by following the leading-edge contour with a dedicated tool.*

## 4 Discussion

Some key industrial sectors (e.g., energy, aerospace, construction, oil and gas), well known for their high capital investment and long service life, rely on various postproduction treatments (e.g., inspection, maintenance, repair) to sustain their businesses. Most of the time, due to scale constraints, it is imperative to perform the postproduction operations *in situ* (Dong et al., 2019). The constrained or dangerous nature of this working environments (e.g., work performed at high altitude or under water, with chemical exposure, in restrained spaces or in explosive environments) raises serious health and safety concerns and calls for some form of robotic service support. This work lays the groundwork for the development and implementation of a deliberative framework aiming to orchestrate the automation of the behavioral process of robotic platforms to be deployed in harsh environments, considering an extended set of functional capabilities (navigation, perception of human activities, HRI, emotion recognition, planning and execution of MRO tasks).

Three underlying behavioral dimensions could be further addressed when considering the functional capabilities instantiated with the support of the *Safety* and *MRO HRI* behavior trees: ascertaining the current working situation involving the human, in particular to identify potential hazards; grasping the emotional state of the human (in addition to physical difficulties of handling certain tasks, an excessive human psychological burden could lead to irreversible losses); reasoning and proposing alternative routes to alleviate the difficulty of tasks to be handled by the human, through shared human-robot plans.

### 4.1 Understanding Dangerous Situations Involving Human

Robotic solutions capable of performing common field operator tasks in hazardous and unpleasant working environments can bring significant benefits towards the improvement of the health and safety in the workplace environment. However, when it comes to operation in harsh environments, a substantial lack of robotic system autonomy has been noted, either due to the complexity of the problems to be solved or as a consequence of an insufficient maturity level of involved technologies and systems, lacking reliability and robustness where tasks demand high success rates (Wong et al., 2018). Furthermore, with constrained spaces and highly-contaminated facilities, fully autonomous solutions are unlikely to be considered safe or cost-effective in the near future, although there is a significant interest in the development of such capabilities (Bandala et al., 2019). In this respect, the advent of more efficient sensors, electronics and algorithms enables under certain constraints the ability of robotic platforms to recognize dangerous scenarios in harsh environments (Alon et al., 2021; De Fazio et al., 2021). Within our BT framework, this recognition is enabled through context data acquisition and interpretation across two channels 1) through the multimodal health state classification relying upon the output of the embedded sensors (e.g., evaluate human’s posture, gesture, facial expression, current emotional state; sense frustration, distress, work overload or possible injury situation) and 2) through direct communication with the human (e.g., ask what type of activities needs to be completed, what type of support is needed, check human availability to perform specific tasks). Subsequently, an additional context assessment model is required to put together the information and decide if the data from both channels is consistent. This task is partially handled by the compound risk assessment phase proposed in this work, although a comprehensive evaluation could be carried out only by implementing a *Theory Of Mind* (Devin and Alami, 2016) functionality, which would endow the robot with the capability to simulate the outcomes of a shared plan and to determine the consequences of the planned actions. This would promote an enhanced level of confidence about the evaluations performed to validate both the current situation (e.g., perception of a non-ergonomic posture of the human which might lead to injuries) and future scenario.

### 4.2 Grasping Human Emotional State

One thing that makes us truly human is the ability to empathize. Since the emotional interactions are a crucial way for humans to understand each other, it comes naturally to expect the autonomous technology to adopt the very same human traits in its interaction with us. However, designing robotic platforms with our exact emotionality should not necessarily be the goal (Malinowska 2021) and a more pragmatic and functional approach is desired for empathy modelling. In this respect, the underlying principle proposed by our framework is the understanding of human emotional responses in relation with various working contexts and the formulation of adapted robotic behaviors aiming to alleviate the perceived human stress factors and to reduce the human exposure to dangerous situations.

Recently, the interest in automated human emotion recognition and its practical implementation in the HRI context is steadily increasing and finds more areas of application (Dzedzickis et al., 2020; Spezialetti et al., 2020). The availability of relevant models and methods with various maturity levels provides a sound background for the development and implementation of empathy-like robotic behaviors within our framework. The occurrence of such states during HRI might arise at specific points in time during a mission, in particular associated with the deliberative nodes of the proposed BT structure. In this respect, the timely evaluation of the current situation guides the subsequent robot’s behavior and its actions across the following tasks. Two different semantics could be associated with the behaviors deployed by the robot to complete a mission: *aware* and *non-aware*. For instance, if the robot is programmed to execute an MRO task with a particular teammate, it means it is aware of the ID of the teammate and it will adapt its navigation, grounding, interaction and deliberation behaviors, i.e. follow and execute the *Safety HRI* routine, with respect to the identified teammate. A non-aware behavior occurs for instance when the risky working behavior of a human, not related with the robot’s initial mission, might catch the attention of the robot if their trajectories intersect themselves. In this respect, further ground needs to be covered to endow the deliberative nodes with the capability to seamlessly handle the arising conflicts between the aware/non-aware situations.

### 4.3 Reasoning and Re-Planning

Robots can be repaired, but people cannot, when serious injuries occur. We can argue this statement alone is strong enough to justify the takeover of all hazardous activities by robots. However, in order to accommodate this role change, we need to shed some light on the autonomous reasoning and planning skills of the robot. The enhancement of these capabilities in harsh environments relies on the fulfillment of two major requirements: 1) the robot is able to receive mission goals and to automatically sequence skills to achieve them, and 2) the robot is able to update its initial plans in the presence of unknown events based on its contextual perception.

In order to address the first requirement, an initial understanding and a comprehensive planning of the activities and procedures involved in a shared human-robot MRO process is required. The establishment of an empathy map could significantly contribute to gaining more insight into the whole process and a more profound and diverse problem understanding including also the emotional needs and feelings of human teammates. The downstream analysis of the empathy map supports the profiling of the hazardous steps in the *as-is plan*, i.e., the conventional approach of completing an MRO task, exclusively performed by the humans. Next, the identification of potential candidates relies upon the assessment of the task risk level imposed on three different categories in the perspective of a partial or complete transfer of the risk from the human to the robot. However, the planning of shared activities is not trivial, since the intrinsic task hazard reduction through robot takeover could lead to a complete procedural change of the working routine. For instance, supporting preparation and/or safety surveillance activities related to repair and maintenance processes, as well as the human resources required to support their realization, could become redundant (Heilemann et al., 2021).

In this context, any task configuration activity must be addressed considering the outcomes of a prior interdependence analysis as well as the availability of suitable process, control and validation steps. Furthermore, the redesign of a task for collaborative purposes must be fully informed when it comes to the accurate definition of the course of action for each agent involved. This can be realized by giving full consideration to the adopted task planning formalism while considering the action type, action time-line as well as the synchronous, sequential or independent execution of the activities (Tan et al., 2010; Johannsmeier and Haddadin 2017; Buisan et al., 2020).

As far as the second requirement is concerned, it is worthwhile mentioning that, depending on the nature of collaboration, minor execution errors can impact in different ways the performance of the initial shared tasks planning. If the human teammate works too slowly or makes mistakes, the timing and successful completion of the robot’s actions may be impacted. Very much alike, if the robot’s timing is off or performs outside of its tolerance specifications, a human teammate or another robot may be required to compensate. In this respect, the framework must be endowed with interfaces to facilitate the role switching between the teammates, as otherwise the team performance might degrade. Furthermore, each teammate should know the capabilities, needs and weaknesses of others and should be able to understand and predetermine their actions. From the robot’s perspective, the integration of several monitoring nodes aiming to signal sudden changes in the self-state, in the human’s behavior and on-the-job emotional state, or in the task environment, could enable the existing BT framework to anticipate next teammate actions, to suggest a course of action to the teammates or to possibly ask them for help if necessary.

## 5 Conclusion

During the last years a sheer number of applications for mobile robot systems have emerged across various sectors. Yet the technology has so much more to offer in the matter of human-robot interaction. The main contribution of this paper is to present a deliberative framework aiming to orchestrate the automation of the behavioral process of robotic platforms to be deployed in harsh environments. The proposed architecture, based on the behavior tree formalism, enables the organization of an extended set of robotic capabilities (navigation, perception of human activities, HRI, emotion recognition, deliberation, planning and execution of MRO tasks) centered on the provision of adapted behavioral responses to improve the occupational health and human welfare.

The concept and the initial formulation of the robotic behaviors are greatly simplified, as many of them are indeed challenging research problems in their own right. Any complete solution would need to handle a wider search space of behaviors as well as a larger collection of robotic platforms. The elaboration of a first framework for the MRO sector and the employment of the behavior tree formalism allows us to start with simpler initial implementations of the individual tasks and then gradually replace them with more complex models (e.g., through machine learning) and extend them to other platforms, without changing the structure of the initial tree, or the implementation of the prior tasks.

## Data Availability

The original contributions presented in the study are included in the article/Supplementary Material, further inquiries can be directed to the corresponding author.
